# Deferiprone targets aconitase: Implication for Friedreich's ataxia treatment

**DOI:** 10.1186/1471-2377-8-20

**Published:** 2008-06-16

**Authors:** Sergio Goncalves, Vincent Paupe, Emmanuel P Dassa, Pierre Rustin

**Affiliations:** 1 Inserm, U676, Hôpital Robert Debré, Paris, F-75019 France and Université Paris 7, Faculté de Médecine Denis Diderot, IFR02, Paris, France

## Abstract

**Background:**

Friedreich ataxia is a neurological disease originating from an iron-sulfur cluster enzyme deficiency due to impaired iron handling in the mitochondrion, aconitase being particularly affected. As a mean to counteract disease progression, it has been suggested to chelate free mitochondrial iron. Recent years have witnessed a renewed interest in this strategy because of availability of deferiprone, a chelator preferentially targeting mitochondrial iron.

**Method:**

Control and Friedreich's ataxia patient cultured skin fibroblasts, frataxin-depleted neuroblastoma-derived cells (SK-N-AS) were studied for their response to iron chelation, with a particular attention paid to iron-sensitive aconitase activity.

**Results:**

We found that a direct consequence of chelating mitochondrial free iron in various cell systems is a concentration and time dependent loss of aconitase activity. Impairing aconitase activity was shown to precede decreased cell proliferation.

**Conclusion:**

We conclude that, if chelating excessive mitochondrial iron may be beneficial at some stage of the disease, great attention should be paid to not fully deplete mitochondrial iron store in order to avoid undesirable consequences.

## Background

Friedreich ataxia (FRDA) is a severe neurological disease with progressive cerebellar ataxia associating cardiomyopathy. It originates from a triplet expansion in the first intron of the gene coding for frataxin and the resulting impaired transcription causes depletion of this mitochondrial protein [1]. The actual consensus states that frataxin function, through the handling of mitochondrial iron, is intimately related with the synthesis of iron-sulfur clusters (ISC) subsequently distributed to the various cell compartments [2]. A number of ISC containing enzymes play a crucial role in cell metabolism. In keeping with this, aconitase was found severely affected in Friedreich ataxia and its residual activity might be a crucial issue to determine the course of the disease [3]. Indeed, while the activity of the mitochondrial enzyme is determinant for the metabolic flux through the tricarboxylic acid in the mitochondria [4], the cytosolic counterpart of the aconitase is known to tightly regulate the overall iron metabolism of mammal cells [5]. Accordingly, loss of aconitase activity has been previously shown to trigger cell death of cardiac fibroblasts [6]. Beside the impaired ISC assembly, an increased susceptibility to oxidative insult [7] and a late mitochondrial iron accumulation [8] have been reported as a result of frataxin depletion. Therefore, both antioxidant- and mitochondrial iron chelator-based therapies have been initially considered [9]. However, while antioxidants were readily trialled with some promising results [10], iron-chelator therapy could not be initially assessed because desferrioxamine, the only chelator in widespread clinical use at that time, did not target mitochondrial iron [11]. Recently the interest in using an iron chelation approach was renewed due to the availability of deferiprone, a chelator specifically targeting mitochondrial iron [12,13]. In this context, we studied the *in vitro *effect of deferiprone in control and FRDA patient cultured skin fibroblasts and in a shRNA frataxin-depleted neuroblastoma-derived cell line (SK-N-AS cells).

## Methods

Fibroblasts derived from forearm biopsies taken with informed consent from healthy controls and two FRDA patients (10–20% residual frataxin mRNA in their fibroblasts) were grown under standard conditions in Dulbecco's modified Eagle's medium (DMEM; Gibco Invitrogen, Cergy Pontoise, France) supplemented with 10% foetal calf serum, 10 mg/ml penicillin/streptomycin and 2 mM L-Glutamine (as Glutamax™; Gibco Invitrogen). Final iron content in culture medium amounted to 2–3 μM. The medium (4 ml/25 cm^2 ^flask; 3 ml/10 cm^2 ^well) was changed each three days. Fibroblasts were seeded at 18 × 10^3 ^cells/cm^2^. SK-N-AS cells, seeded at 150 × 10^3 ^cells/cm^2^, were grown in the same culture medium added with 100 μM non essential amino acid mixture (Gibco Invitrogen) and 200 μM uridine (Sigma-Aldrich, St Quentin, Falavier, France). Fibroblasts were treated with 25, 75 or 150 μM deferiprone for 7 days. SK-N-AS cells were treated for a variable duration (0, 1, 7 days) with 150 μM deferiprone final. The deferiprone effect on these cells was in addition tested for 7 days using 10, 50, 150 μM of the drug. Cell counting was done after trypsination using the Quick Read Precision Cell (Globe Scientific Inc., NJ, USA).

For the sake of comparison between cell types, SK-N-AS cells derived from a neuroblastoma were also used after frataxin silencing using shRNA. In brief, frataxin-depleted SK-N-AS were obtained by transducing lentiviral particles carrying a gene encoding for frataxin-directed shRNA and a puromycin resistance cassette (MISSION™ TRC shRNA; Sigma-Aldrich, St Louis, Missouri USA). Frataxin-targeting sequence of the shRNA was CCGGGCTGGACTCTTTAGCAGAGTTCTCGAGAACTCTGCTAAAGAGTCCAGCTTTTT. SK-N-AS cells were seeded in 24-well plates. After 24 h, 2 μg/ml hexadimethrine bromide was added just before infection with lentiviral particles. After 18 h, cells were washed 3 times with PBS. Successfully infected cells were selected with 10 μg/ml puromycin (15 d).

Aconitase (EC 4.2.1.3) measurement was spectrophotometrically carried out by following aconitate production from citrate at 240 nm [3] on the supernatant (800 *g *× 5 min) of detergent-treated cells (0.2% lauryl maltoside). All chemicals were of the purest grade available from Sigma-Aldrich (St Quentin; Falavier, France). Protein concentration was measured according to Bradford method.

Data are shown as mean ± 1 SE. Measurements were analyzed by *t *test. All statistical tests were calculated using SigmaStat software (Sigma, St Louis, USA); *p *< 0.001 was considered to indicate statistical significance and denoted by *** for each value; *n.s. *non significant.

## Results

### Deferiprone gradually abolishes aconitase activity in human cultured cells

**Figure 1:**
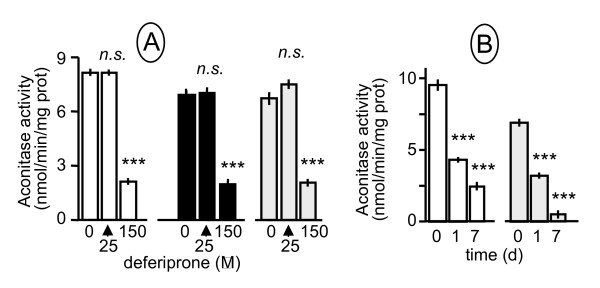
**Deferiprone targets aconitase enzyme**. Aconitase inhibition triggered by deferiprone in (**A**) cultured skin fibroblasts from control (open square) and two Friedreich's ataxia patients after 7 d of culture. Time-dependent aconitase inhibition by 150 μM deferiprone in (**B**) control (open square) and frataxin-depleted (shRNA treated) SK-N-AS cells. Error bars correspond to 1 SE (n = 3); *** denotes *p *< 0.001; n.s. non significant. Experimental conditions as described under Methods.

The activity of cytosolic and mitochondrial aconitase, two ISC containing proteins, is known to be highly dependent on iron availability [14]. We thus first investigated in human cultured skin fibroblasts the capacity of the enzymes to resist the iron depletion resulting from a deferiprone treatment (Fig. 1A). Mitochondrial aconitase activity represents up to 80% of the total aconitase activity measurable in these cells. We observed that 150 μM deferiprone treatments resulted in a significant and progressive loss of aconitase activity (up to 60% loss after 7 days) in control and patient's fibroblasts. An even more pronounced loss of aconitase activity was induced by 150 μM deferiprone in frataxin-depleted and non-depleted SK-N-AS cells (Fig. 1B). Deferiprone-induced loss of aconitase activity was both dose- and time-dependent (Fig. 1). Noticeably, 150 μM deferiprone had no effect on the activity of aconitase when added during enzyme assay (not shown), indicative that the loss of aconitase activity observed in cells should be ascribed to the chelation of available iron rather than to a direct effect of the chelator on the ISC of the enzyme.

### Deferiprone inhibits growth of human cultured cells

**Figure 2:**
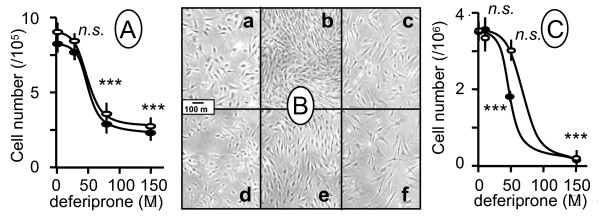
**Deferiprone decreases cell proliferation**. Effect of 7 d-treatment with deferiprone (0–150 μM) on (**A**) control (open symbol) and patient fibroblasts and on (**C**) control (open symbol) and frataxin-depleted SK-N-AS cells. A light microscope view (**B**) (×4) of control (a, b, c) and patient (d, e, f) fibroblasts, before treatment (18 h after seeding; a, d), or 7 d in the absence (b, e) or presence (c, f) of 150 μM deferiprone. Error bars correspond to 1 SE (n = 3); *** denotes *p *< 0.001; *n.s. *non significant. Experimental conditions as described under Methods.

We next investigated the consequences of the loss of aconitase activity induced by 150 μM deferiprone (7 d) on cell proliferation (Fig. 2). Not surprisingly in view of the critical role of theses enzymes in cell metabolism [14], we observed that the severe loss of aconitase activity was concomitant with a major impairment of cell growth of control and patient's fibroblasts (Fig. 2A, B) or of control and frataxin-depleted SK-N-AS cells (Fig. 2C).

## Discussion

The results reported in this study confirm that deferiprone is a potent chelator of mitochondrial matrix iron [12]. As such, it also potently impairs aconitase activity, presumably through reduced synthesis of the iron-sulfur cluster machinery which makes use of available iron in the mitochondrial matrix with the help of frataxin [15]. Conflicting results have been reported concerning mitochondrial iron content of FRDA patient's fibroblasts which may or not accumulate iron [16]. We show here that the abnormal status of mitochondrial iron in Friedreich ataxia possibly at the origin of the hypersensitivity of these cells to oxidative insults – associated or not with an increased mitochondrial iron content – does not protect frataxin depleted cells from the deleterious effect of iron chelation. So far we have no idea of the concentration of deferiprone which might be reached in brain tissues of treated patients. Nevertheless, a mean peak plasma concentration of deferiprone has been estimated to be about 150–200 μM when deferiprone is provided in one daily dose of 25 mg/kg of body weight [17]; a value similar to the 150 μM of deferiprone used in this *in vitro *study. A lower concentration is probably maintained in tissues, however if deferiprone concentration is efficient to empty mitochondrial iron it should also gradually impair aconitase activity in the mitochondrial matrix. Obviously, the activity of additional iron-requiring enzymes might be affected as well due to excessive lowering of mitochondrial iron, e.g. the mitochondrial ribonucleotide reductase [18]. Assaying the iron-sensitive activity of these mitochondrial enzymes might provide useful maker(s) for iron chelator toxicity in future clinical studies.

## Conclusion

Consequently, while iron chelation might help the mitochondrion to cope with excessive iron at some particular step of the disease, *i.e. *when iron accumulation is instrumental, its putative efficacy on a long term will predictably be associated with a detrimental mitochondrial iron deprivation. Ultimately, this might further impair the mitochondrial synthesis of ISC, already a critical issue in Friedreich ataxia.

## Competing interests

The authors declare that they have no competing interests.

## Authors' contributions

SG conceived and performed the experiments. VP generated and studied the frataxin-depleted SK-N-AS cells. ED characterized the chelator-treated cells and interpreted the results. PR conceived the study and wrote the manuscript.

## Pre-publication history

The pre-publication history for this paper can be accessed here:


